# A predictive scoring system for proximal junctional kyphosis after posterior internal fixation in elderly patients with chronic osteoporotic vertebral fracture: A single-center diagnostic study

**DOI:** 10.3389/fendo.2022.923778

**Published:** 2022-07-22

**Authors:** Xing Du, Guanyin Jiang, Yong Zhu, Wei Luo, Yunsheng Ou

**Affiliations:** ^1^ Department of Orthopedics, The First Affiliated Hospital of Chongqing Medical University, Chongqing, China; ^2^ Orthopedic Laboratory of Chongqing Medical University, Chongqing, China

**Keywords:** osteoporotic vertebral fracture, posterior internal fixation, proximal junctional kyphosis, elderly, prediction, scoring system

## Abstract

**Objective:**

To establish a predictive scoring system for proximal junctional kyphosis (PJK) after posterior internal fixation in elderly patients with chronic osteoporotic vertebral fracture (COVF).

**Materials and methods:**

The medical records of 88 patients who were diagnosed with COVF and underwent posterior internal fixation in our hospital from January 2013 to December 2017 were retrospectively analyzed. The included patients were divided into two groups according to whether they suffered PJK after surgery, namely, the PJK group (25 cases) and non-PJK group (63 cases). The following clinical characteristics were recorded and analyzed: age, gender, body mass index (BMI), bone mineral density (BMD), smoking history, fracture segment, proximal junction angle, sagittal vertebral axis, pelvic incidence (PI)–lumbar lordosis (LL), pelvic tilt (PT), sacral slope (SS), posterior ligamentous complex (PLC) injury, upper instrumented vertebra, lower instrumented vertebra, and the number of fixed segments. The prevalence of these clinical characteristics in the PJK group was evaluated, and the scoring system was established using logistic regression analysis. The performance of the scoring system was also prospectively validated.

**Results:**

The predictive scoring system was established based on five clinical characteristics confirmed as significant predictors of PJK, namely, age > 70 years, BMI > 28 kg/m^2^, BMD < −3.5 SD, preoperative PI-LL > 20°, and PLC injury. PJK showed a significantly higher score than non-PJK (7.80 points *vs.* 2.83 points, *t*=9.556, *P*<0.001), and the optimal cutoff value for the scoring system was 5 points. The sensitivity and specificity of the scoring system for predicting postoperative PJK were 80.00% and 88.89%, respectively, in the derivation set and 75.00% and 80.00% in the validation set.

**Conclusion:**

The predictive scoring system was confirmed with satisfactory sensitivity and specificity in predicting PJK after posterior internal fixation in elderly COVF patients. The risk of postoperative PJK in patients with a score of 6–11 is high, while the score of 0–5 is low.

## Introduction

Osteoporotic fracture (OF) is a worldwide clinical challenge. Osteoporotic vertebral fractures (OVFs) are the most common form of OF and often occur in the thoracolumbar vertebrae of the elderly (1). OVF with a course of more than 3 months is defined as chronic osteoporotic vertebral fractures (COVFs) (2). The early-stage clinical symptoms of elderly COVF patients are not obvious, but with the increase of age, the degree of osteoporosis and the kyphosis are progressively aggravated, resulting in intractable lumbago pain and even delayed paralysis, which seriously affect the life quality of patients (3). The clinical efficacy of conservative treatment for elderly COVF is rarely satisfactory (4). At present, the main treatment of elderly COVF is posterior long segmental internal fixation, which can effectively maintain the spine stability, correct the kyphosis, and reduce the risk of fracture vertebral collapse and kyphosis progression (5).

However, the risk of proximal junction kyphosis (PJK) after posterior long segment internal fixation is high with the reported incidence of 6%–40% because the thoracolumbar spine is located at the junction of spinal force line transmission (6). Although most PJK patients have mild clinical symptoms, severe PJK patients may develop into proximal borderline failure (PJF), or even neurological impairment, and consequently require a revision operation (7). Furthermore, most PJK patients are elderly people, they have poor bone condition and many medical complications; thus, the risk of PJK revision surgery is really high (8). Therefore, in the treatment of elderly COVF by posterior long segmental internal fixation, it is of great significance to actively detect the risk factors of PJK.

Although two studies have reported the risk factors for PJK (6, 7), they had limited guiding implications for clinical work due to the totally different risk factors reported by them. In addition, the two studies did not focus on elderly patients. Thus, the risk factors for postoperative PJK in elderly COVF are remain controversial, and further studies are still needed.

Therefore, in this research, we hypothesized that the ability of predicting PJK after posterior internal fixation in aged COVF can be enhanced by establishing a scoring system *via* investigating the risk factors of PJK after surgery.

## Materials and methods

This study was approved by the Ethics Committee of the First Affiliated Hospital of Chongqing Medical University (2017-97). All of the participants provided their written informed consent to participate in this study. The work has been reported in line with the STARD criteria (9).

### Patient selection

**Figure 1:**
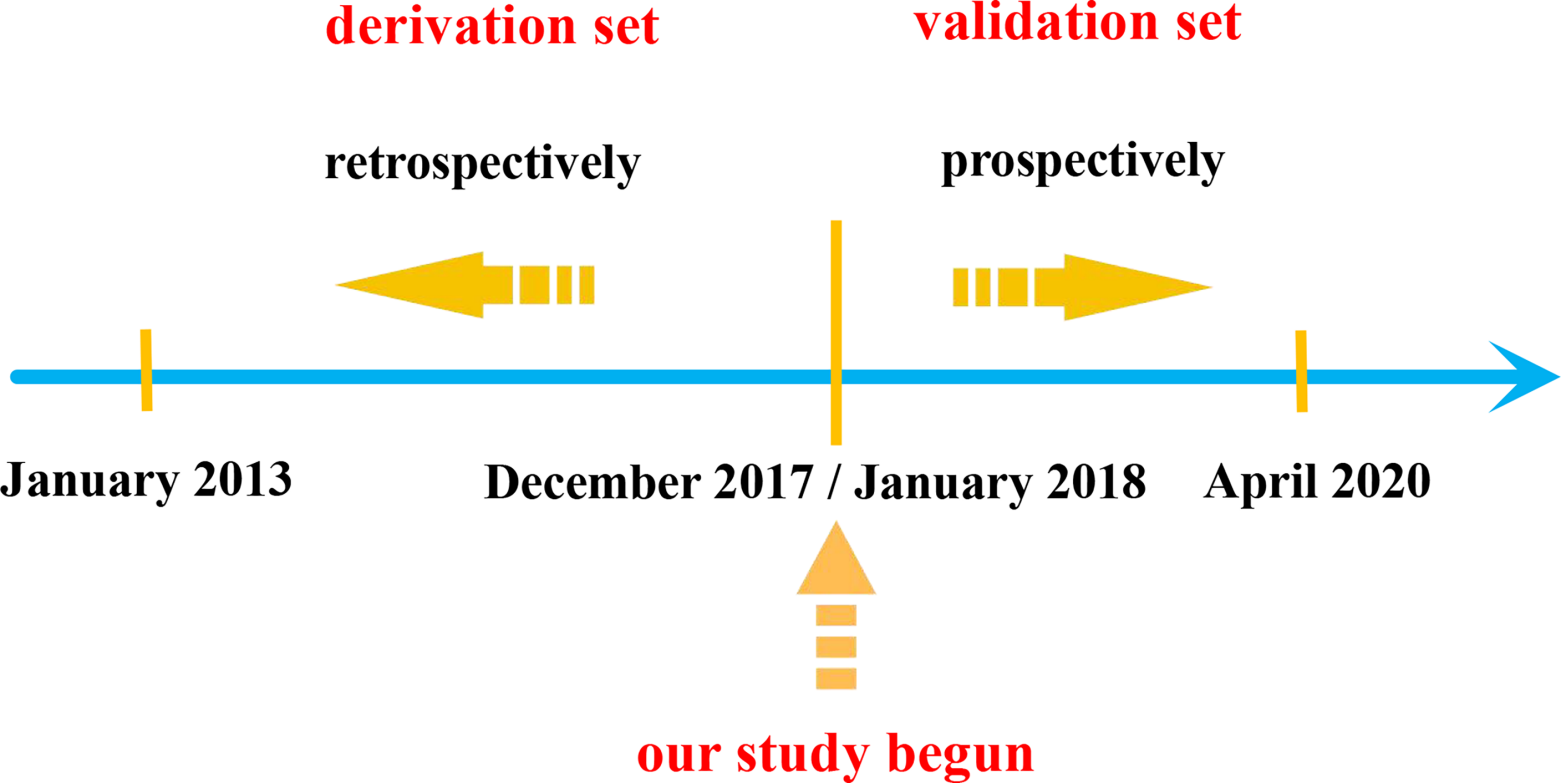
Schematic of patient inclusion in derived and validation sets in this study.

We retrospectively reviewed the medical records of hospitalized patients diagnosed with COVF in our department from January 2013 to December 2017 to form the derivation set (**Figure 1**).

Inclusion criteria: (1) The diagnosis was single-segment COVF; (2) age ≥ 60 years old; (3) preoperative CT showed that the posterior wall of the vertebral collapses and protrudes into the spinal canal but without exceeding 1/3 of the spinal canal; (4) patients who underwent posterior long segments internal fixation (≥5 segments); (5) patients treated with conventional non-operative treatment for more than 3 months but no significant improvement in symptoms; and (6) bone density showed T value ≤ −2.5 standard deviation (SD).

Exclusion criteria: (1) Previous history of spinal surgery or severe spinal cord injury; (2) patients with idiopathic or congenital spinal deformity, spinal tumor, infection, or tuberculosis; (3) pathological vertebral fracture; (4) lower limb surgery history, which may affect imaging data measurement; and (5) less than 12 months follow-up or incomplete medical record data.

### Surgical procedure

All surgeries were performed by experienced spinal surgeons in the same medical group. After general anesthesia, the patient was placed in a prone position with a sponge pad placed under the chest and pelvis to make the abdomen suspended. After disinfection and towel spreading, a C-arm X-ray machine was used to locate the fractured vertebra, and then, the posterior median incision was made with the kyphosis vertex as the center. Next, the paravertebral muscles were separated, and at least two normal vertebrae were exposed with the injured vertebrae as the center. The vertebral and its upper and lower articular processes of the fixed segments were exposed, and pedicle screws were inserted into two vertebrae above and below the fractured vertebrae. For patients with severe osteoporosis, the fixation segment can be appropriately extended and the channel of pedicle screws can be strengthened with bone cement, mainly strengthening one-to-two groups of proximal and distal screws. Then, single-segment pedicle subtraction osteotomy (PSO) was performed to correct the kyphosis. Firstly, the bone rongeur was used to remove the spinous process, lamina, and bilateral pedicle of the osteotomy vertebrae. Next, a short titanium rod was used alternately to temporarily fix the upper and lower adjacent segments of the osteotomy vertebrae. Then, a short titanium rod alternate was used to temporarily fix the adjacent sections of the vertebrae, and then, the vertebral vertebra and lower vertebral vertebra were removed for spinal canal decompression, the nerve root was revealed and protected and finally placed on the connection of both sides, and the screws were tightened one by one. The parietal vertebra and the lamina of the upper and lower vertebrae were then removed for spinal canal decompression, with care taken to expose and protect the nerve roots. Finally, prebent connecting titanium rods were placed on both sides and the nuts were tightened one by one. After C-arm X-ray fluoroscopy verified the satisfactory correction of kyphosis and the examination of no active bleeding, the wound was flushed, the drain was placed, and the surgical incision was closed layer by layer.

### Data collection

Based on the results of previous studies and our experience, we included the possible following predictors for posterior PJK, which mainly included the patient- related data, preoperative imaging data, and surgery-related data.

**Figure 2:**
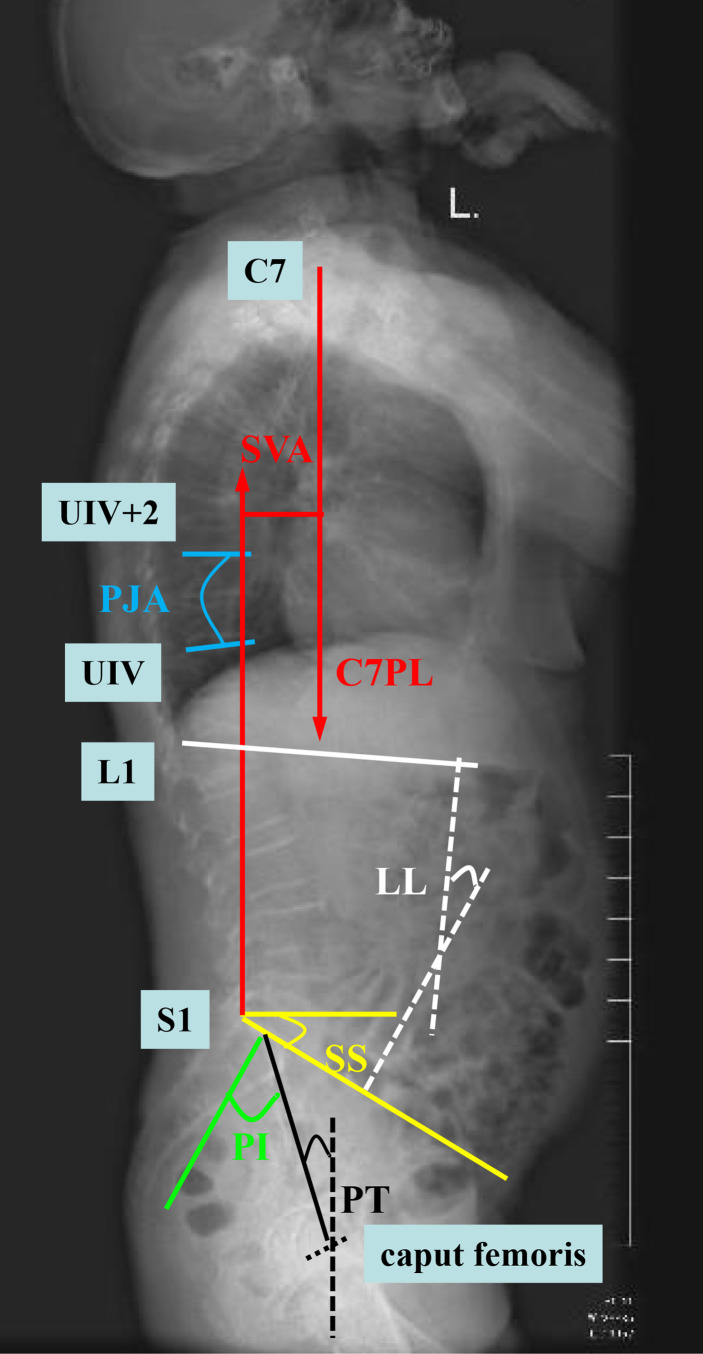
Diagram of the measurement of imaging data. SVA (sagittal vertebral axis; red line), PJA (proximal junction angle; blue line), LL (lumbar lordosis; white line), PI (pelvic incidence; green line), PT (pelvic tilt; black line), and SS (sacral slope; yellow line).

(1) Patient-related data: (a) age ≥70 years. (b) The gender was female. (c) Body mass index (BMI) > 28 kg/m^2^. (d) The T-value of bone mineral density (BMD) < -3.5 SD. (e) Had a smoking history. (f) The fracture segment was T_12_ or L_1_ vertebrae.

(2) Preoperative imaging data (**Figure 2**): (a) proximal junction angle (PJA) > 5°: PJA was the angle between the lower endplates of the upper instrumented vertebra (UIV) and upper endplates of the second distal vertebrae of the UIV (UIV+2). (b) sagittal vertebral axis (SVA) > 50 mm: SVA was the vertical distance between the C7 plumb line and the posterior upper angle of S1. (c) Pelvic incidence (PI)—lumbar lordosis (LL) > 20°: PI was the angle between the line A and B. Line A is between the midpoint of the S1 endplate and the midpoint of the line that connects the center of two femoral heads. Line B is the perpendicular of the S1 upper endplate passing through the midpoint of the S1 endplate. (d) Pelvic tilt (PT) > 30°: PT was the angle between the plumb line and the straight line between the midpoint of the S1 endplate and the midpoint of the line that connects the center of two femoral heads. (e) Sacral slope (SS) > 25°: SS was the angle between the S1 endplate and the horizontal line. (f) posterior ligamentous complex (PLC) injury: PLC injury was a single or combined injury of the supraspinous ligament, interspinous ligament, and ligamentum flavum, which may be accompanied by facet fracture.

(3) Surgery-related data: (a) the UIV location was T_10_ to T_12_ vertebrae. (b) The lower instrumented vertebra (LIV) was S_1_ vertebrae. (c) Number of fixed segments > 7.

(4) Follow-up outcomes: postoperative PJK was defined as postoperative PJA ≥ 10° and increased by more than 10° compared with preoperative. The final follow-up time was 2 years after surgery.

### Development of the scoring system

Firstly, all the included patients were divided into two groups, namely, the PJK group and non-PJK group according to the 2-year postoperative follow-up outcomes. Secondly, univariate analysis was conducted on the patient-related data, preoperative imaging data, and surgery-related data of the two groups. Based on the results of univariate analysis, the index with *P*<0.05 was considered a possible predictor for postoperative PJK. Next, multivariate logistic regression analysis was performed for the indexes with *P*<0.05 in univariate analysis. According to the results of multivariate logistic regression analysis, the indexes with *P*<0.05 were considered the final predictors for postoperative PJK and, thus, determined as the items of the scoring system. Then, we established the weighted score of each item based on the relative size of the odds ratio (OR) according to the method reported by our previous research (10). Finally, we made the appropriate cutoff points for the scoring system using receiver operator characteristic receiver operator characteristic (ROC) curves corresponding to the point on the curve nearest the upper left corner of the ROC graph.

### Validation of the scoring system

From January 2018 to April 2020, we prospectively included patients to validate the accuracy of the scoring system (**Figure 1**).

The following criteria were used to determine whether a patient should be prospectively included in the validation set. The inclusion criteria were as follows: (1) elderly patients (age ≥60 years) who were preoperatively diagnosed with single-segment COVF. (2) Preoperative bone density showed that the T-value ≤ −2.5 SD. (3) Patients who have the surgical indication. The exclusion criteria were as follows: (1) a previous history of spinal surgery, lower limb surgery, and severe spinal cord injury; (2) patients with idiopathic or congenital spinal deformity, spinal tumor, infection, or tuberculosis; and (3) pathological vertebral fracture.

The patients included in the study signed informed consent and then underwent long posterior segmental fixation surgery. After surgery, the spinal surgeon reviewed the patient’s clinical data and calculated the score according to the scoring system and then predicted whether this patient will suffer from PJK (defined as the predictive outcome). At the follow-up of 2 years after surgery, the included patient was assessed whether they actually developed PJK (defined as the final follow-up outcome). The accuracy of the scoring system was evaluated by comparing the consistency between the predictive outcome and the final follow-up outcome.

### Statistical analysis

The clinical characteristics were subjected to univariate logistic regression analysis, and the significant factors were evaluated by multivariate logistic regression analysis. The items of the scoring system were determined by multivariate logistic regression, and the weighted score of each item was based on the relative size of the OR. The optimal cutoff point was made by using ROC curves. *P* < 0.05 was the set of statistical significance. The SPSS version 10.0 software was used for statistical analysis.

## Results

### Derivation of the scoring system

A total of 88 patients were included in the derivation set, including 25 cases in the PJK group and 63 cases in the non-PJK group, and the incidence of postoperative PJK was 28.41%.

Univariate analysis showed that age > 70 years, BMI > 28 kg/m^2^, BMD < −3.5 SD, preoperative PJA > 5°, preoperative SVA > 50 mm, preoperative PI-LL > 20°, PLC injury, UIV location = T_10_~T_12_, LIV location = S_1_, and the number of fixed segments > 7 were the risk factors of postoperative PJK (**Table 1**).

**Table 1:** Univariate analysis of related variables of predicting postoperative proximal junctional kyphosis (PJK).

Variables	PJK group (N=25)	Non-PJK group (N=63)	Sensitivity (%)	Specificity (%)	*P*-value
Gender = Male	10/25	19/63	40.00	69.84	0.376
Age > 70 years	16/25	8/63	64.00	87.30	<0.001
BMI > 28 kg/m^2^	17/25	8/63	68.00	87.30	<0.001
BMD < −3.5 SD	19/25	13/63	76.00	79.37	<0.001
Smoking history	9/25	22/63	36.00	65.08	0.924
Fracture segment =T_12_ or L_1_	18/25	40/63	72.00	36.51	0.448
Preoperative PJA > 5°	12/25	15/63	44.00	76.19	0.026
Preoperative SVA > 50 mm	15/25	22/63	60.00	65.08	0.032
Preoperative PI-LL > 20°	17/25	16/63	68.00	74.60	<0.001
Preoperative PT > 30°	21/25	43/63	84.00	31.75	0.135
Preoperative SS > 25°	11/25	29/63	44.00	53.97	0.863
PLC injury	19/25	14/63	76.00	77.77	<0.001
UIV location = T_10_~T_12_	17/25	22/63	68.00	65.08	0.005
LIV location = S_1_	16/25	23/63	64.00	63.49	0.019
Number of fixed segments > 7	16/25	25/63	64.00	60.31	0.039

PJK, proximal junctional kyphosis; BMI, body mass index; BMD, bone mineral density; SD, standard deviation; PJA, proximal junction angle; SVA, sagittal vertebral axis; PI, pelvic incidence; LL, lumbar lordosis; PT, pelvic tilt; SS, sacral slope; PLC, posterior ligamentous complex; UIV, upper instrumented vertebra; LIV, lower instrumented vertebra.

Multivariate logistic regression analysis was carried out on the significant findings in univariate analysis and showed five clinical characteristics, namely, age > 70 years, BMI > 28 kg/m^2^, BMD < −3.5 SD, preoperative PI-LL > 20°, and PLC injury were significant predictors of postoperative PJK (**Table 2**).

**Table 2:** Multivariate analysis of related variables of predicting postoperative PJK.

	Regression coefficient (β)	Odds ratio (OR)	*P*-value
Age > 70 years	3.16	23.57	<0.001
BMI > 28 kg/m^2^	2.03	7.61	0.022
BMD < −3.5 SD	3.08	21.76	<0.001
Preoperative PI-LL > 20°	2.55	12.81	0.019
PLC injury	2.60	13.46	0.014

We developed a scoring system based on these five clinical characteristics that were conformed significant predictors of postoperative PJK. The variables with a significant predictive value for postoperative PJK were given the weighted scores according to the relative value of the OR in multivariate logistic regression analysis: age > 70 years, BMI > 28 kg/m^2^, BMD < −3.5 SD, preoperative PI-LL > 20°, and PLC injury were weighted as 3 points, 1 point, 3 points, 2 points, and 2 points, respectively. The score was then calculated by determining the total number of points, ranging from 0 to 11 (**Table 3**).

**Table 3:** The scoring system for predicting postoperative PJK.

Variables	Score
Age > 70 years	
Yes	3
No	0
BMI > 28 kg/m^2^	
Yes	1
No	0
BMD < −3.5 SD	
Yes	3
No	0
Preoperative PI-LL > 20°	
Yes	2
No	0
PLC injury	
Yes	2
No	0

**Figure 3:**
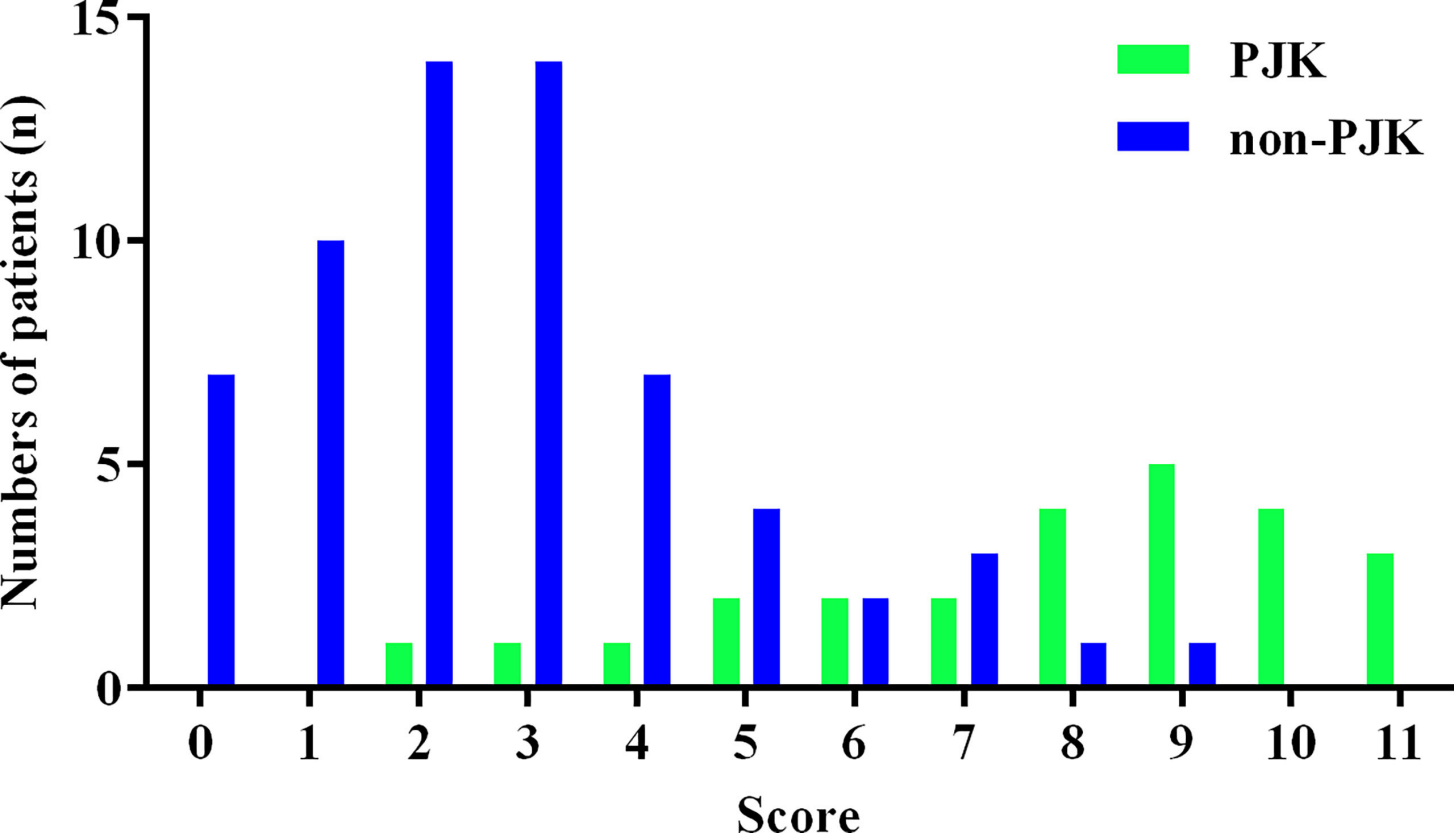
Histogram distribution of proximal junctional kyphosis (PJK) and non-PJK for each score of the predictive scoring system.

**Figure 4:**
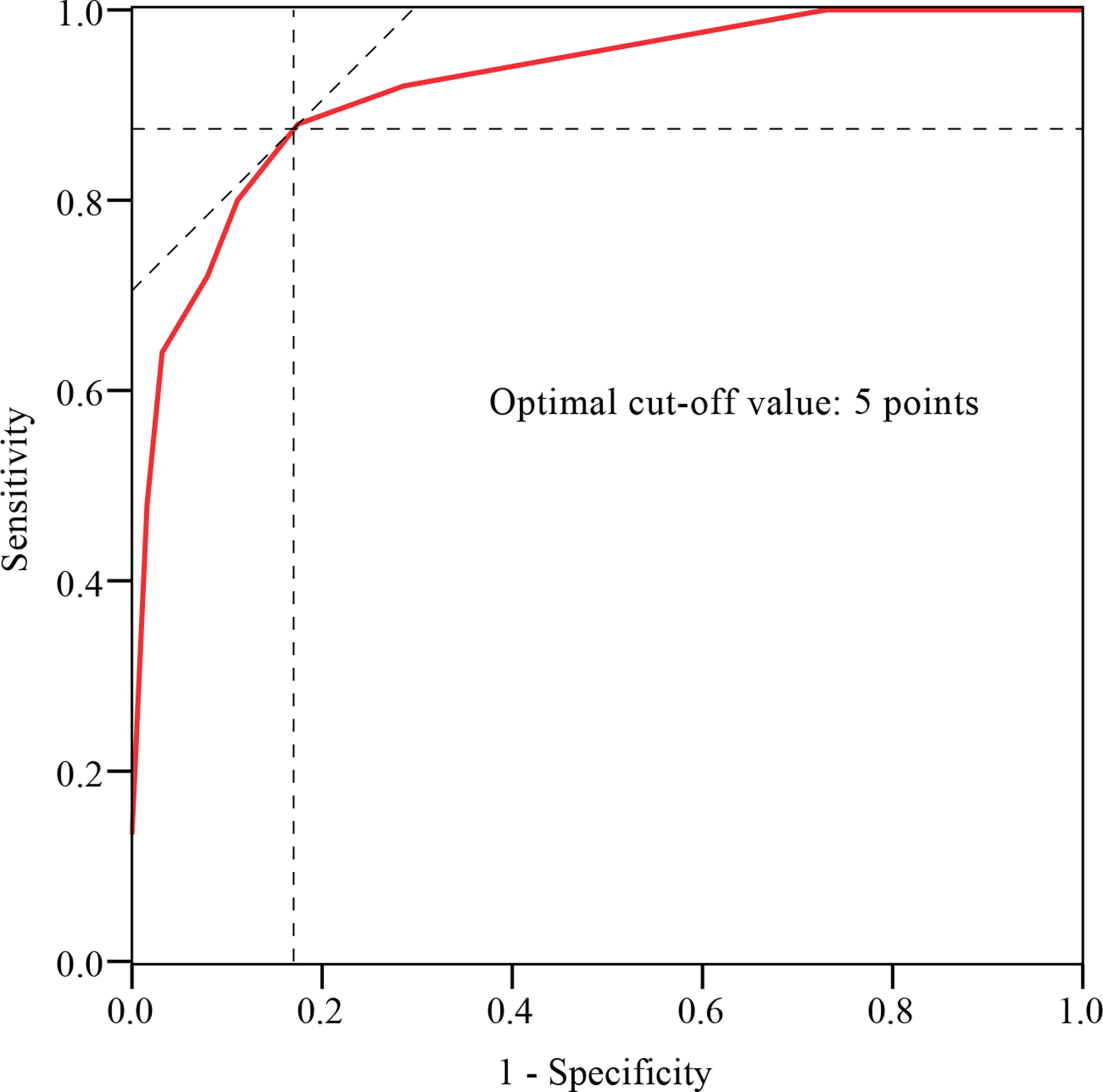
ROC curve analysis of the predictive scoring system. The optimal cutoff point based on the ROC curve analysis of scores was 5 points.

The histogram distribution of the score values is shown in **Figure 3**. Remarkably, the PJK group showed a significantly higher score than the non-PJK group (7.80 points *vs.* 2.83 points, *t*=9.556, *P*<0.001). The optimal cutoff value of the predictive scoring system was 5 points, and the area under the curve (AUC) was 0.921 (95% CI: 0.875–0.985, *P<*0.001) (**Figure 4**).

### Validation of the scoring system

Finally, a total of 42 patients were prospectively included in the validation set, including 12 cases in the PJK group and 30 cases in the non-PJK group according to the 2-year postoperative follow-up outcomes. A comparison of the performance of the score system on the derivation set and validation set is shown in **Table 4**. Based on the cuoff value of 5 points, the sensitivity and specificity of the scoring system for predicting postoperative PJK were 80.00% and 88.89%, respectively, in the derivation set and 75.00% and 80.00% in the validation set.

**Table 4:** Comparison of performance of the scoring system on derivation set and validation set.

		Derivation set			Validation set		
		PJK (score ≥ 6)	Non-PJK (score ≤ 5)	Total	PJK (score ≥ 6)	Non-PJK (score ≤ 5)	Total
Outcomes	PJK	20	5	25	9	3	12
	Non-PJK	7	56	63	6	24	30
	Total	27	61	88	15	27	42
Sensitivity (%)		80.00			75.00		
Specificity (%)		88.89			80.00		

## Discussion

### Risk factors of proximal junctional kyphosis after surgery

In our present study, age >70 years was found a risk factor of postoperative PJK. A previous study reported that PJK was more likely to occur when people are over 55 years old, and the risk of PJK increased with age (11). Kim et al. and Yang et al. also found that PJK had a higher incidence in people whose age was over 60 years (12, 13). The reasons may be as follows: (1) in patients with spinal deformity, degenerative changes may occur in paravertebral muscle tissue over time; (2) the degeneration of the paravertebral muscles can cause uneven stress in the discs and spinal facet joint, which can also accelerate the degeneration of the spine. Moreover, advanced age was also considered to be an important risk factor for revision surgery for PJK (7).

This study concluded that BMI >28 kg/m^2^ predicted a high risk of postoperative PJK, and this conclusion was similar to the previous study. Previous research reported that patients with BMI >25 kg/m^2^ were prone to suffer from PJK after surgery (14), which may be due to the following reasons: (1) the obese patient has a heavier load on the spine and implants, and the weight of the body moves forward, resulting in increased stress on adjacent segments of the surgery (15); (2) in obese patients, the strength of the paravertebral muscle was significantly weakened, and the dissection of the lamina and spinous muscles may further affect the muscle function and ultimately accelerate the proximal joint degeneration (16).

In this study, BMD < −3.5 SD was confirmed an independent risk factor for PJK after surgery. O’Leary et al. also showed that osteoporosis patients were more prone to develop PJK because the reduction of bone mass and the destruction of the bone ultrastructure can reduce the screw-holding force and increase the risk of the screw loosening and pulling out (17). Moreover, decreased bone mineral density was associated with decreased paravertebral muscle tissue, which, together, may lead to spinal instability and accelerate the development of PJK (18).

This study found that preoperative PI-LL > 20° was an independent predictor of postoperative PJK, which was similar to the result of the previous study. PI-LL was an important imaging index reflecting whether the lumbar lordosis angle was compatible with the shape of the pelvis, indicating the compensatory state of the sagittal balance of the spine (19). Senteler et al. found that higher PI-LL would increase the compression force and shear force of the L_3_~L_5_-moving segments, leading to the accelerated degeneration of adjacent vertebral segments, thus increasing the risk of PJK (20). Aoki et al. found that when the preoperative PI-LL was between 10° and 20°, patients could obtain better clinical efficacy and the incidence of postoperative PJK was lower (21).

The results of our study suggested that PLC injury was an independent risk factor for postoperative PJK, and this conclusion was similar to a previous study (22). Surgery may change the local anatomy and biomechanics of the spine, leading to the development of PJK (23). For example, posterior spinal surgery may cause damage to proximal soft tissues, including supraspinal and intermuscular ligaments, and spinal facet joint capsule injuries, which may lead to local stability loss and PJK (24).

### Managements to reduce the risk of postoperative proximal junctional kyphosis

Protective measures for patients’ own factors mainly include: (a) lumbar back muscle function exercise. With the increase of a patients’ age, paravertebral muscle atrophy and fat infiltration are serious, leading to the decline of paravertebral muscle strength, which is closely related to the occurrence of postoperative PJK (25). Therefore, appropriate muscle function training can help reduce the risk of PJK. (b) Lose weight. Weight loss can reduce the physical stress in the muscles and bones of the proximal junction, thereby reducing the risk of postoperative PJK (26). (c) Anti-osteoporosis treatment. Standard anti-osteoporosis treatment can improve bone calcium content and bone strength, which is conducive to maintain the stability of the spinal internal fixation system and reduce screw loosening and pulling out (27).

The imaging-related factors affecting postoperative PJK were mainly sagittal sequence reconstruction. Therefore, a preoperative full measurement and analysis of spinal imaging data and the intervention of high-risk groups by identifying high risk factors are of great significance to reduce the risk of PJK after surgery. We suggest that, according to the sagittal evaluation criteria and sagittal spinal sequence score of adult spinal deformity formulated by the Scoliosis Research Society (SRS) (28, 29), a reasonable surgical plan should be formulated to properly correct the deformity and take into account the overall balance of the spine.

Protective measures for surgery-related factors mainly include: (a) soft tissue protection. When exposing the distal vertebral region, attention should be paid to the protection of the muscle–ligament complex to minimize the damage to the supraspinal and interspinous ligaments. The separation of the paraspinal muscles at the junction should be carefully handled to retain the ligament structure and muscle attachment of the midline to the maximum extent (30). (b) The enhancement of ligaments in the junction area. Ligamentous augmentation by tendon transplantation or silk reinforcement can reduce the stress at the junction and increase the strength of PLC (31). (c) Non-rigid fixation (32). The use of lamina hooks in the proximal fixation area provides a relatively non-rigid fixation structure that helps to protect adjacent segmental facet joints and intervertebral discs, prevent excessive stress concentration in the junction area, and reduce the occurrence of PJK or PJF.

Our study also has limitations. First, this study was a retrospective analysis research. Second, the sample size was small and the follow-up time was short. Third, other potential factors that may contribute to PJK, such as disease course and comorbidity, were not analyzed in this study.

## Conclusion

The scoring system, which was based on five clinical characteristics, namely, age > 70 years, BMI > 28 kg/m^2^, BMD < −3.5 SD, preoperative PI-LL > 20°, and PLC injury, seems to achieve satisfactory sensitivity and specificity in predicting PJK after posterior internal fixation in elderly COVF patients. The risk of postoperative PJK in patients with a score of 6–11 is high, while the score of 0–4 is low.

## Data availability statement

The raw data supporting the conclusions of this article will be made available by the authors, without undue reservation.

## Ethics statement

The studies involving human participants were reviewed and approved by the Ethics Committee of the First Affiliated Hospital of Chongqing Medical University. The patients/participants provided their written informed consent to participate in this study.

## Author contributions

Conception and design: XD and YO. Data analysis and interpretation: XD, GJ, and YZ. Data collection and management: XD, GJ, and WL. Manuscript writing and critical revisions: all authors. Overall responsibility: XD and YO. All authors have read and approved the manuscript.

## Conflict of interest

The authors declare that the research was conducted in the absence of any commercial or financial relationships that could be construed as a potential conflict of interest.

## Publisher’s note

All claims expressed in this article are solely those of the authors and do not necessarily represent those of their affiliated organizations, or those of the publisher, the editors and the reviewers. Any product that may be evaluated in this article, or claim that may be made by its manufacturer, is not guaranteed or endorsed by the publisher.
